# Application of Chitosan and Boehmite as Ecological Fire Retardants in PVC Compositions—Preparation and Thermal Study

**DOI:** 10.3390/molecules30214222

**Published:** 2025-10-29

**Authors:** Kamil Dziuba, Andrzej Puszka, Katarzyna Dawidek, Beata Podkościelna

**Affiliations:** 1 Department of Organic Chemistry and Crystallochemistry, Institute of Chemical Science, Faculty of Chemistry, Maria Curie-Sklodowska University, Gliniana St. 33, 20-614 Lublin, Poland; kamil.dziuba@mail.umcs.pl; 2 Department of Polymer Chemistry, Institute of Chemical Science, Faculty of Chemistry, Maria Curie-Sklodowska University, Gliniana St. 33, 20-614 Lublin, Poland; andrzej.puszka@mail.umcs.pl (A.P.); katarzyna.dawidek@elpar.pl (K.D.)

**Keywords:** ecological fire-retardants, chitosan, boehmite

## Abstract

Eco-friendly flame retardants are becoming a popular alternative to traditional fire retardants, many of which contain toxic halogens. These modern additives, which are based on phosphorus, nitrogen, or silicon compounds, minimize the emission of harmful gases during combustion, making them safer for the environment and human health. This study aimed to synthesize and analyze poly(vinyl chloride) (PVC) composites using a newly synthesized hybrid fire retardant, boehmite derivative (aluminium dibutyl phosphonate), as an environmentally friendly additive. The fire-retardant properties of chitosan, which is derived from the natural biopolymer chitin, have also been tested. The chemical structure of the synthesized compounds was confirmed using ATR/FTIR spectroscopy and SEM-EDX analysis. Next, PVC-based dry blends were prepared with the addition of a stabilizer, plasticiser, chalk, and selected flame retardants (aluminium dibutyl phosphonate or chitosan) at concentrations of 10 wt%, 30 wt%, and 50 wt%, resulting in homogeneous materials intended for evaluating fire performance, thermal stability (DSC, TGA), and mechanical resistance.

## 1. Introduction

Plastics are widely used across various industries, including construction, electronics, and manufacturing, and are therefore often required to meet stringent fire safety standards. Most organic polymers exhibit limited resistance to fire. To reduce their flammability and ensure compliance with regulatory classifications, formulations are typically modified with flame-retardant additives. These compounds function by delaying ignition, slowing flame propagation, reducing smoke emission, and limiting heat release during combustion [1,2,3].

Flame retardants operate through various physicochemical pathways, broadly grouped into gas-phase and condensed-phase actions, as well as synergistic systems that combine both strategies [4,5]. There are two types of additives: reactive flame retardants and additive flame retardants. Reactive flame retardants are chemicals that contain heteroatoms, providing a degree of flame resistance; additionally, they are chemically integrated into the polymer chains [6]. The most efficient formulations often rely on phosphorus–nitrogen synergy, which couples gas-phase radical quenching with condensed-phase char promotion [7]. In the case of poly(vinyl chloride) PVC, which inherently contains chlorine atoms and possesses moderate flame resistance, flame retardant modifications typically focus on reducing smoke emission, lowering heat release, and improving fire classification under European standards [8].

Environmental concerns and legislative restrictions (REACH, RoHS, WEEE) on halogenated retardants drive the development of halogen-free, non-toxic, and biodegradable solutions. Therefore, current research focuses on several categories of alternative flame retardants: -mineral additives such as magnesium dihydroxide (MDH), aluminum trihydroxide (ATH), boehmite or nanofillers, which act primarily through endothermic decomposition and physical barrier formation [9,10,11,12,13]; -nitrogen, phosphorus–nitrogen compounds, including melamine, APP or melamine phosphate, which participate in char formation and release non-flammable gases [14,15,16]; -bio-based retardants derived from renewable or waste biomass, e.g., lignin, starch, cellulose, chitosan [17,18,19].

Chitosan, derived from chitin, is a promising bio-based flame retardant. Its thermal degradation leads to the formation of a stable, carbonaceous char, which serves as a protective barrier against heat and oxygen. Additionally, the amino functionalities within the chitosan structure contribute to flame inhibition by releasing inert gases such as ammonia, thereby diluting combustible volatiles in the gas phase. Depending on the application, chitosan can be employed either as a matrix additive or as part of surface modification strategies, such as layer-by-layer (LbL) assembly. For instance, Malucelli and co-workers developed LbL coatings comprising chitosan and oxidized graphene for flexible polyurethane foams [19]. These coatings significantly reduced flammability, with reported decreases of 54% in peak heat release rate (pHRR) and 59% in total smoke production. A recent scientometric review highlights the increasing attention given to chitosan-based flame-retardant systems, particularly those incorporating phosphorylated derivatives and aerogel structures. These materials exhibit promising thermal stability and char-forming ability, which can be further enhanced by the inclusion of inorganic additives.

Boehmite (γ-AlOOH), a halogen-free aluminum oxyhydroxide, has gained attention as an effective flame retardant due to its multi-functional role in polymer matrices. Upon heating to approximately 480 °C, it undergoes endothermic dehydration, releasing water and converting into thermally stable alumina (Al_2_O_3_). This transformation not only absorbs heat but also creates a ceramic-like barrier that inhibits oxygen diffusion and limits the evolution of flammable volatiles. Experimental studies confirm the efficacy of boehmite in enhancing flame retardancy. Sun et al. demonstrated that incorporating 10.2 wt% boehmite into epoxy resin increased the limiting oxygen index (LOI) from 18.5% to 37.4%, and raised the decomposition temperatures, indicating improved thermal stability and potential for char formation [13].

Organophosphorus-based flame retardants can effectively enhance the flame resistance of polymer materials with minimal effects on their physical and mechanical properties [20,21,22]. These flame retardants can create a sealed carbonaceous foam layer on the combustion surface, serving as a flame retardant, oxygen barrier, smoke suppressor, and drop inhibitor [23]. Primarily, alkyl and aryl aluminum phosphates represented a class of hybrid nanomaterials or nanofillers in polymer compositions [24,25]. Currently, aluminum organophosphates constitute a novel category of hybrid flame retardants that are effectively employed in the process of production of polymer composites [26,27].

This study aimed to synthesize a hybrid flame retardant—dibutyl aluminum phosphate—using readily available and safe reagents. The structure and properties of the obtained compound were confirmed by using ATR/FTIR, SEM-EDX, and DSC analysis. The new material was tested as an effective flame retardant in PVC composites. In addition, chitosan was examined as a second biopolymer-based, environmentally friendly flame retardant, applied in the same quantities and under identical synthesis conditions. The spectral, thermal, mechanical, and flammability properties of the prepared PVC composites were studied in detail. The use of these components provides an eco-friendly alternative to halogenated flame retardants and aligns with current trends in sustainable, synergistic fire protection technologies that comply with European and international standards.

## 2. Results and Discussion

### 2.1. Modification of Boehmite

Alkyl and aryl aluminum phosphates are a type of hybrid flame retardant that has been effectively used to make polymer composite materials with heat resistance and fire safety [28]. Their use as flame retardants does not affect the mechanical properties of polymer composite materials, which also exhibit excellent resistance to high temperatures and direct flames, and are environmentally safe. For multi-gram synthesis of aluminum dibutyl phosphonate, a new hybrid compound with potential flame-retardant properties, and economic reasons, our research utilized readily available commercial reagents, boehmite (PURAL SB, Sasol, Brunsbüttel, Germany) and dibutyl phosphonate (Merck, Darmstadt, Germany). However, to optimize the preparation of such compounds, the effect of reaction temperature on the degree of conversion, yield, and product structure was investigated. The materials, boehmite and dibutyl phosphate, were heated to the boiling points of aromatic solvents like toluene (110.6 °C), xylene (138–142 °C, a mix of isomers), and mesitylene (164.7 °C), which helps carry out the reaction at high temperatures while also efficiently getting rid of water from the reaction area using an azeotropic distillation technique.

**Figure 1:**
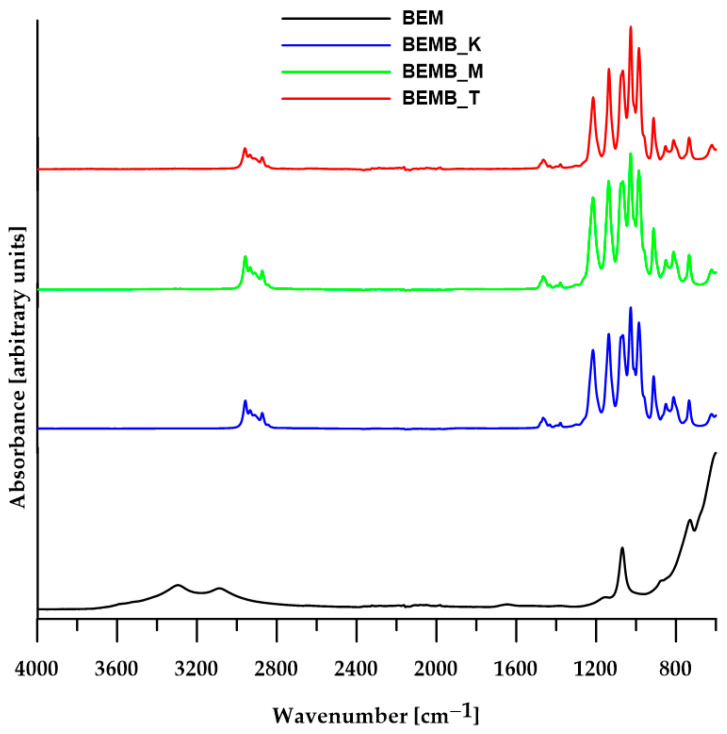
ATR/FT-IR spectra of boehmite (BEM) and aluminum dibutyl phosphates (BEMB_K, BEMB_M and BEMB_T).

For each reaction, regardless of the solvent used, researchers obtained white fine-grained powders with a yield exceeding 80%, which were then subjected to ATR analysis (TENSOR 27, Bruker, Ettlingen, Germany) after drying. Figure 1 presents a comparison of spectra recorded by Fourier Transform Infrared Spectroscopy (FT-IR) for boehmite samples and three pure products obtained from its reaction with dibutyl phosphonate.

The spectrum of pure boehmite shows a clear band for the vibrations of Al-O-Al bonds (610 cm^−1^), a band for the stretching vibrations of the hydroxyl group (3298 and 3085 cm^−1^) and bending vibrations of the Al-OH group (1161, 1068 cm^−1^, and 737 cm^−1^). When products are made by heating boehmite and dibutyl phosphate in different solvents and at various temperatures, the absorption band images of the alumina-phosphate samples look quite alike, even though they are very different from the original materials’ spectra (Figure 1). All the FT-IR spectra of the products contain characteristic absorption bands (2959–2873 cm^−1^) originating from the aliphatic carbon chain, as well as three strong new signals at 1215, 1136, and 1065 cm^−1^, which can be attributed to symmetric and asymmetric stretching vibrations of P-O bonds, confirming the formation of new Al-O-P bonds characteristic of aluminum phosphates. However, it is not possible to accurately assign these peaks to specific types of vibrations due to the coupling of vibrations of the four P-O bonds (two from the Al-O-P-O-Al bridge and two terminal P-O-C bonds) and the lack of ligand geometry. According to the available literature on FT-IR spectra of known aluminum phosphates, the band for symmetric stretching vibrations in the Al-O-P-O-Al bridges appears around 1100 cm^−1^, while the corresponding band for asymmetric vibrations occurs in the range of 1150–1200 cm^−1^.

**Figure 2:**
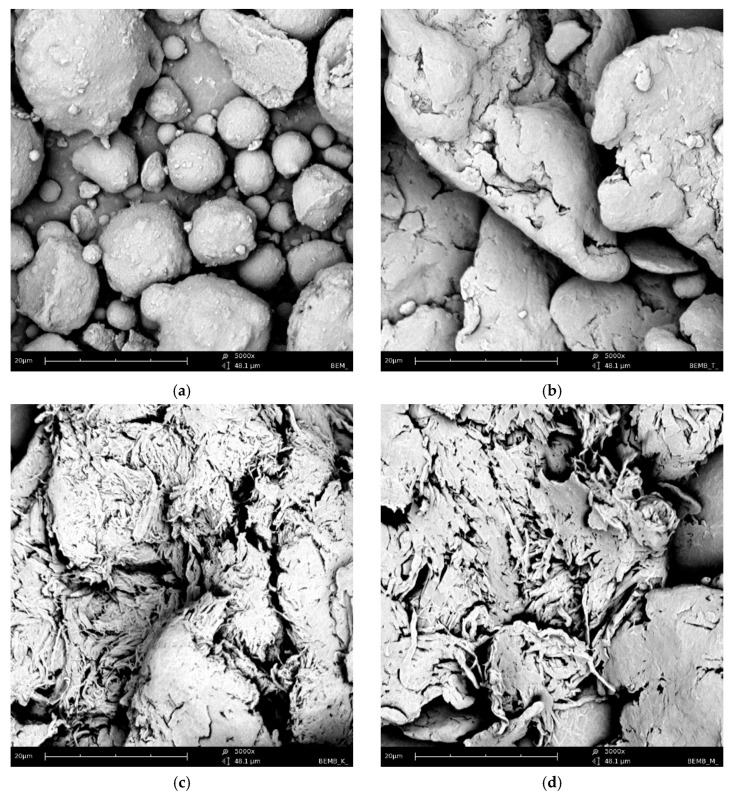
SEM images of: (**a**) boehmite samples (BEM) and aluminum dibutyl phosphates: (**b**): BEMB_T, (**c**): BEMB_K, (**d**): BEMB_M.

**Figure 3:**
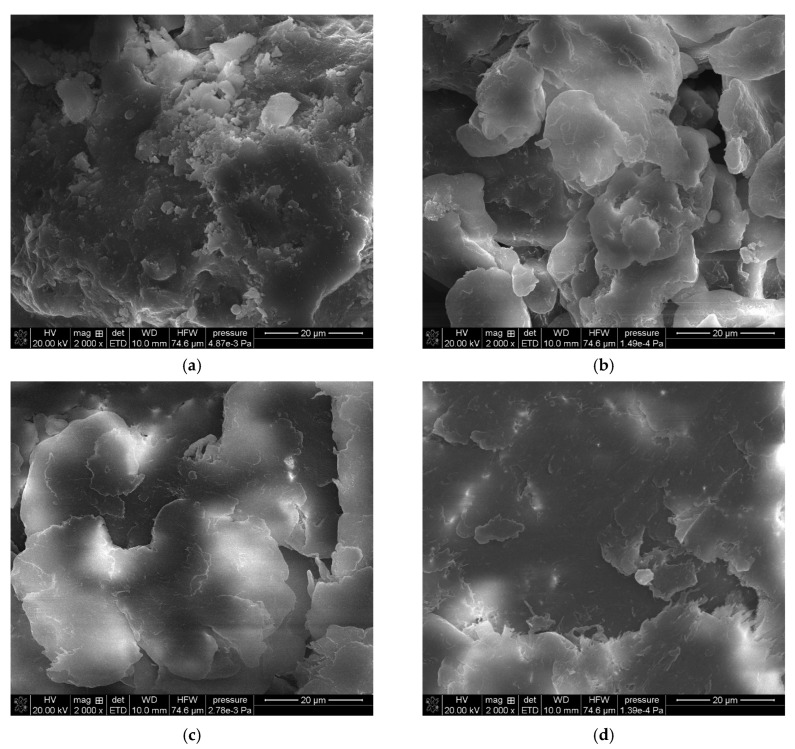
SEM-EDX images of: (**a**) boehmite samples (BEM) and aluminum dibutyl phosphates: (**b**): BEMB_T, (**c**): BEMB_K, (**d**): BEMB_M.

Figure 2 shows a series of images acquired with a scanning electron microscope (SEM Quanta 3D FEG, FEI) for powder samples—boehmite and aluminum dibutyl phosphates obtained in different solvents like toluene, xylene, and mesitylene, along with the reaction conditions based on their boiling points. Significant morphological differences are readily apparent not only when compared to the substrate itself but also between products produced at different temperatures, especially when their synthesis was conducted above 140 °C. The SEM technique also enabled both the identification and measurement of the product sample compositions using energy-dispersive X-ray spectroscopy (EDX), with the images and data shown in Figure 3. Based on the data collected, we can say that the ratio of aluminum to phosphorus atoms measured by the EDX method is about 1:2.2–2.5 for reactions done at temperatures over 140 °C. This means that for every 1 aluminum atom on the surface, there are usually more than 2.1 to 2.5 phospho-organic ligands, and it is starting to resemble the most organized structure, Al(OP)_6_, for this kind of compound (Table 1).

**Figure 4:**
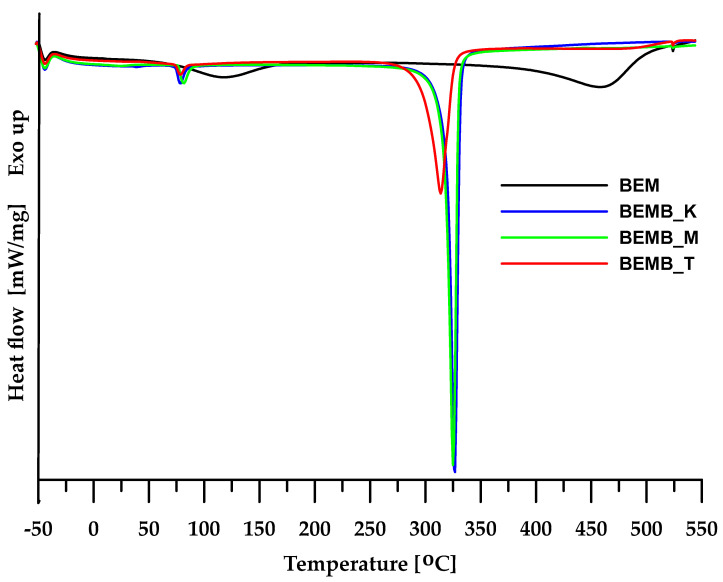
DSC curves of boehmite (BEM) and aluminum dibutyl phosphates (BEMB_K, BEMB_M, and BEMB_T).

For the materials obtained, a DSC analysis was carried out, allowing the determination of the physical changes occurring in the sample during heating. As seen in Figure 4, in the case of pure boehmite, the DSC curve reveals two endothermic peaks. The first peak at 117.6 °C is associated with the evaporation of physically bound water from the sample, while the second peak (at around 458 °C) can be attributed to the transformation of boehmite into γ-Al_2_O_3_. In the case of dibutyl phosphates of aluminum, additional studies also revealed two endothermic peaks, but at different temperature ranges than boehmite. This provides a basis for concluding that the planned modification was successful and the intended product was obtained. The DSC curves of the compounds generated in mesitylene and xylene show a slight difference in their shape compared to the compound obtained in toluene. This suggests that the higher the temperature at which the process is conducted, the more structurally homogeneous the final product becomes. A more detailed analysis of the changes occurring in the material obtained would require additional structural studies, including, among others, crystallographic analysis.

The efficient and technically simple method of synthesizing dibutyl phosphinate of aluminum presented in this work, a new compound from the group of alkyl phosphonates of aluminum, broadens the way for broader applications of this type of hybrid material as thermally resistant flame retardants for composite materials and will undoubtedly be an interesting subject for further research. Thanks to its specific structure and physicochemical properties, it can not only increase the fire resistance of materials but also improve their thermal and mechanical stability in traditional or polymer nanocomposites. Organic analogs of dibutyl phosphinate will soon become an alternative solution to commonly used halogenated flame retardants due to regulations and standards limiting their use. Moreover, the use of such compounds aligns with the trend of seeking more environmentally friendly and safer additives for plastics.

For the materials obtained, a DSC analysis was carried out, allowing the determination of the physical changes occurring in the sample during heating. As seen in Figure 4, in the case of pure bauxite, the DSC curve reveals two endothermic peaks. The first peak at 117.6 °C is associated with the evaporation of physically bound water from the sample, while the second peak (at around 458 °C) can be attributed to the transformation of boehmite into γ-Al_2_O_3_ [28].

**Figure 5:**
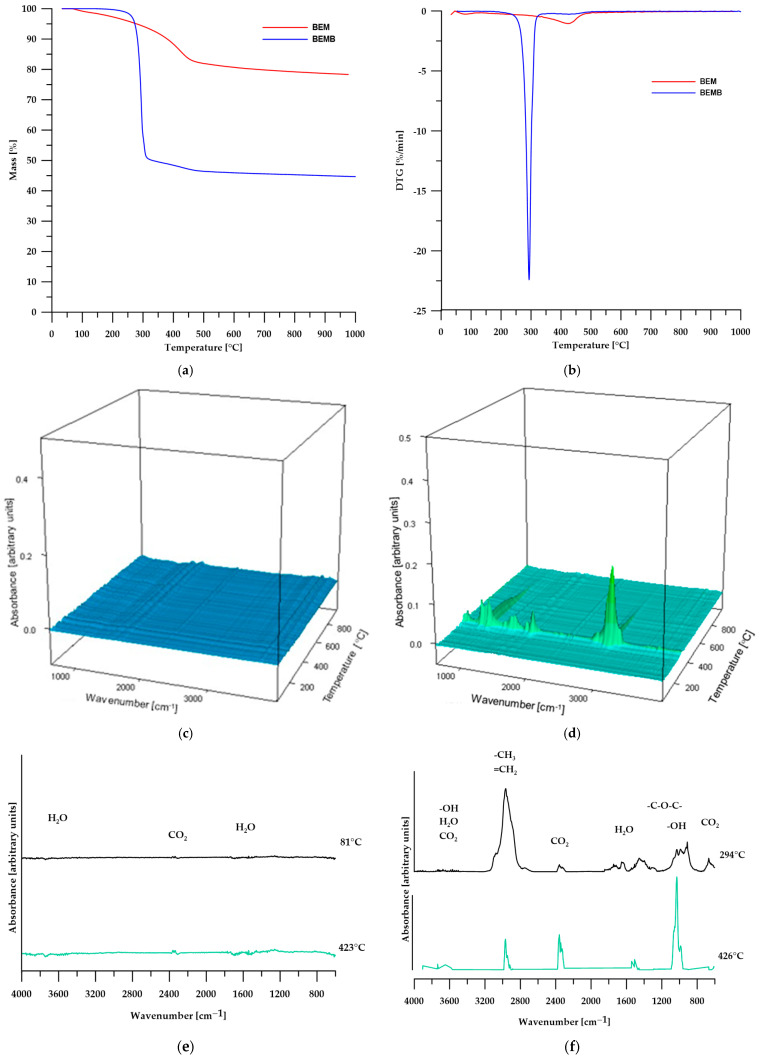
TG (**a**) and DTG (**b**) curves, 3D plots (**c**,**d**) and FT-IR spectra (**e**,**f**) of volatile products obtained during the thermal decomposition.

The TG analysis conducted and confirmed the conclusions drawn from the DSC analysis. Table 2 presents the numerical data from the TG analysis, while Figure 5 shows the TG curves (Figure 5a), DTG curves (Figure 5b), 3D images of the gaseous decomposition products (Figure 5c,d), as well as FT-IR spectra of the gaseous decomposition products of pure boehmite (Figure 5e) and its modification (Figure 5f).

According to the TG analysis of pure boehmite, in the first stage of decomposition (temperature around 81 °C), physical water is desorbed from the sample, while the second stage (temperature around 423 °C) is also associated with the elimination of water, which results from the transformation of boehmite into aluminum oxide according to the following equation:

(1) $$2\;\gamma \text{-AlOOH }\to \;\gamma \text{-}{\mathrm{Al}}_{2}O_{3}+H_{2}O$$

The 3D images and FTIR spectra of the gaseous decomposition products confirm that the only volatile products of boehmite decomposition are water and carbon dioxide, which account for 21.67% of the sample’s mass. The resulting aluminum oxide constitutes 78.33% of the initial mass of boehmite [28,29,30].

In the case of modified boehmite, the DTG curve also revealed two maxima, with the first one (at a temperature of 294 °C) being significantly more intense than the second. This suggests that the highest decomposition rate of the sample occurs at this temperature. The FT-IR spectra of the gaseous decomposition products (Figure 5f) indicate that, in addition to water, the decomposition of BEMB releases aliphatic compounds, alcohols, ethers, and a greater amount of carbon dioxide than in the case of pure boehmite. At a temperature of over 426 °C, water becomes the predominant decomposition product, with only small amounts of aliphatic compounds and carbon dioxide still detectable.

### 2.2. PVC and PVC Composites

**Figure 6:**
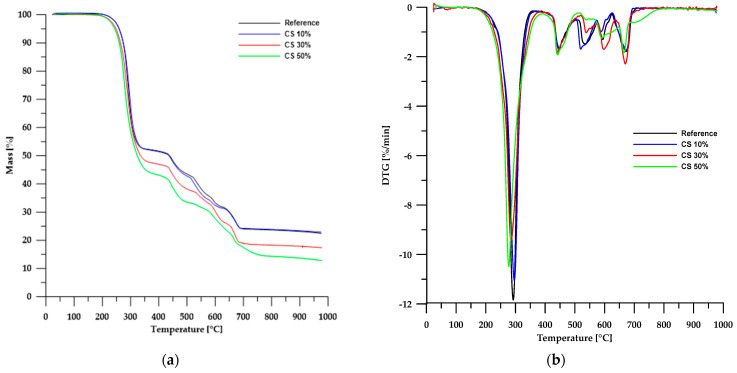
TG (**a**) and DTG (**b**) curves of the CS-based composites.

**Figure 7:**
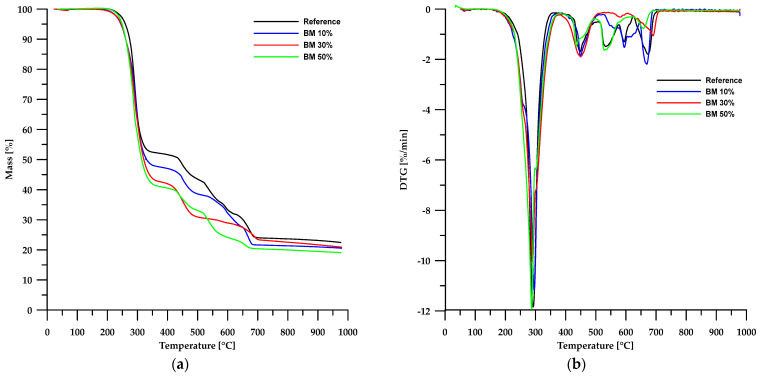
TG (**a**) and DTG (**b**) curves of the BEMB-based composites.

The results of the thermal stability analysis using the TG method of the obtained materials are presented in Table 3, while Figure 6 and Figure 7 show the TG and DTG curves. According to the data in Table 3, the thermal stability of the obtained composites is lower than that of the reference sample and depends on the type of filler used. In the case of materials based on chitosan, an increase in its content in the sample led to a decrease in the stability of such composites. An opposite trend can be observed when modified boehmite is used as a filler.

Partial replacement of chalk with chitosan and modified boehmite resulted in a reduction of the solid residue left in the crucible after analysis. This confirms a decrease in the amount of CaO formed as a decomposition product of chalk. In the case of chitosan, for a composite containing 50 wt% chitosan, the residue amount decreased by approximately 45%, whereas for a corresponding sample with modified boehmite, the residue amount decreased by about 17%. These differences stem from the differences in the composition of chitosan and modified boehmite.

As is known, chitosan is a polysaccharide that decomposes into simple chemical compounds, and depending on the process conditions, its decomposition may additionally generate a small amount of residue in the form of carbon. In the case of boehmite, as previously mentioned, its decomposition produces a deposit in the form of aluminum oxide, which remains in the measuring vessel.

Thermogravimetric analysis of PVC-containing composite materials revealed a multi-stage degradation process, as confirmed by TG and DTG curves (Figure 6 and Figure 7). This complex decomposition pattern indicates the intricate structure of both the reference sample and the composite materials examined.

The first stage of degradation occurs at a *T_max_* of approximately 292 °C and is associated with dehydrochlorination, which involves the elimination of HCl molecules from the PVC polymer chain [31,32,33]. The second stage (*T_max_* ≈ 448 °C) comprises rearrangement reactions and coupling of aromatic structures, leading to the formation of new chemical bonds and further transformation of the polymer structure. The final stages of decomposition take place at temperatures above 520 °C, mainly involving the degradation of inorganic fillers, such as calcium carbonate (CaCO_3_), commonly used as an enhancing component [34,35,36].

A comparison of TG and DTG curves for composites based on chitosan and modified boehmite reveals subtle differences in the initial stages of thermal decomposition. Composites containing CS exhibit a degradation pattern nearly identical to that of the reference sample, indicating similar thermal behaviour. In contrast, materials incorporating modified boehmite display an additional minor DTG peak with a *T_max_* around 256 °C, which may be attributed to the decomposition of the phosphorus-containing ligand present within the material’s structure.

**Figure 8:**
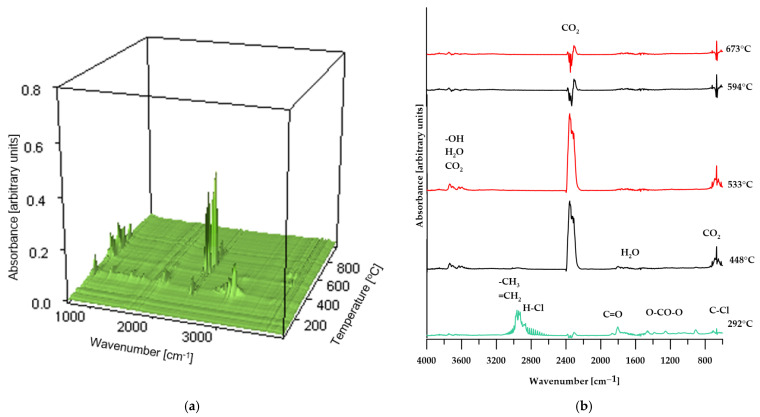
3D plot (**a**) and FT-IR spectra (**b**) of volatile products obtained during the thermal decomposition of the reference sample.

**Figure 9:**
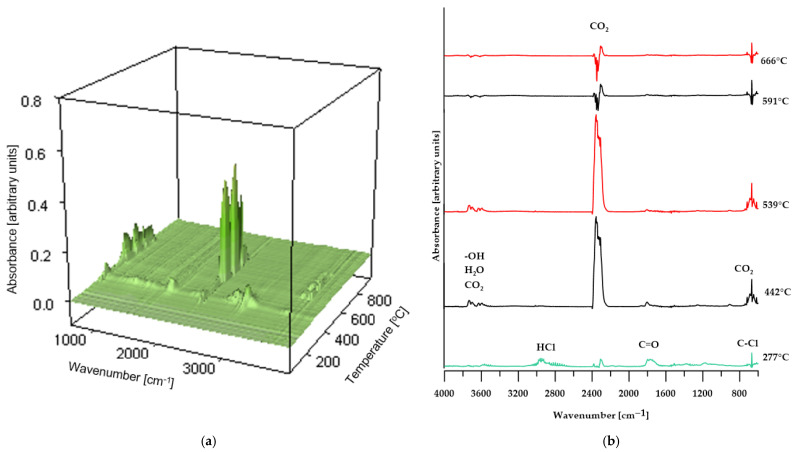
3D plot (**a**) and FT-IR spectra (**b**) of volatile products obtained during the thermal decomposition of sample CS 50%.

**Figure 10:**
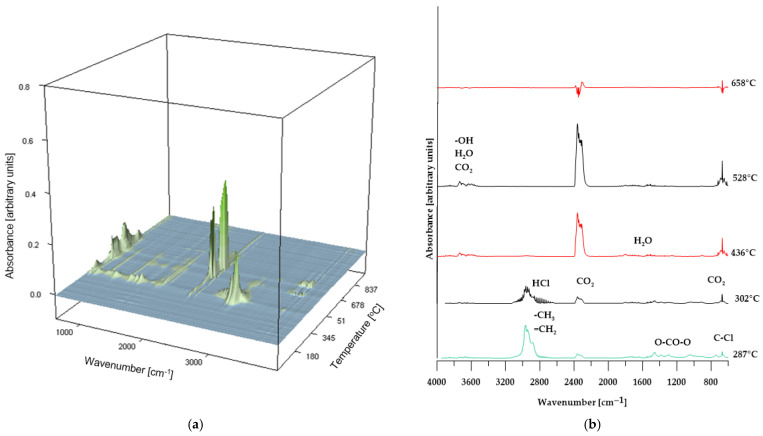
3D plot (**a**) and FT-IR spectra (**b**) of volatile products obtained during the thermal decomposition of sample BM 50%.

To validate the proposed conclusions, the identification of volatile degradation products was carried out, and the resulting spectra are presented in Figure 8, Figure 9 and Figure 10.

As shown in Figure 8, the FT-IR spectra of the volatiles obtained from the decomposition of PVC changed gradually as the temperature increased. At the initial stage of thermal decomposition of the reference sample (maximum temperature of 292 °C), the infrared spectrum (Figure 8a) reveals characteristic absorption bands corresponding to asymmetric stretching vibrations of H–Cl bonds, observed in the range of 3100–2600 cm^−1^. Additionally, the band near 680 cm^−1^ indicates the presence of C–Cl bonds in the structure of the volatile decomposition products, confirming that dehydrochlorination of the sample occurs at this temperature [31,37]. Further absorption bands, associated with the stretching vibrations of carbonyl groups (C=O), the O=C=O group in carbon dioxide molecules, and OCOO-type structures, indicate the release of decomposition products from the plasticizer used in the sample’s production. At elevated temperatures (above 448 °C), the absorption bands characteristic of HCl, halogen-containing compounds, and phthalates disappear. At the same time, there is an increase in the intensity of the absorption band near 2400 cm^−1^, attributed to O=C=O vibrations in CO_2_ molecules; additionally, bands associated with the presence of water molecules appear. Above a temperature of 594 °C, the intensity of the bands corresponding to O=C=O stretching vibrations in CO_2_ decreases and stabilizes for the remainder of the decomposition process [38].

These spectral results support the hypothesis presented earlier in the paper regarding the decomposition mechanism of poly(vinyl chloride).

In the case of composites containing 50 wt% of CS and 50 wt% of BM, the spectra of gaseous decomposition products (shown in Figure 9 and Figure 10, respectively) exhibit strong similarity within comparable temperature ranges. This suggests that no additional or unexpected chemical compounds are released during the thermal degradation of these materials.

However, for the BM 50% sample, the FT-IR spectrum of the gaseous decomposition products (Figure 10b) at 287 °C displays only low-intensity bands typical of dehydrochlorination reactions of PVC. This observation indicates that the dehydrochlorination process in this material has only just begun and reaches its peak at a slightly higher temperature.

**Figure 11:**
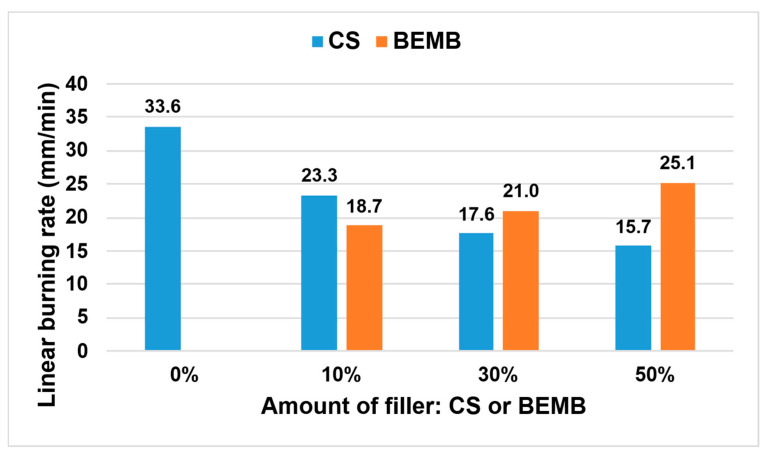
Linear burning rate values of the tested materials.

The thermal properties of the developed materials were assessed based on the analysis of linear burning rate, with the results presented in Figure 11.

The incorporation of chitosan and modified boehmite led to a significant reduction in flame propagation velocity compared to the reference sample. It was observed that increasing the concentration of CS within the composite structure resulted in a progressive decrease in this parameter—already with CS addition, a reduction of more than 50% was recorded relative to the reference material. In contrast, materials containing modified boehmite demonstrated an opposite trend, where higher filler content correlated with increased burning rate. Considering the structure of the applied flame-retardant fillers (CS and BEMB), it was determined that the presence of amino groups—and thus nitrogen atoms—within the chitosan molecule exerted a stronger flame-inhibiting effect than the phosphorus atoms present in the modified boehmite. The findings indicate that partial replacement of chalk with a mixture of chitosan and modified boehmite at 10 wt% significantly enhances the fire resistance of the composite material.

**Figure 12:**
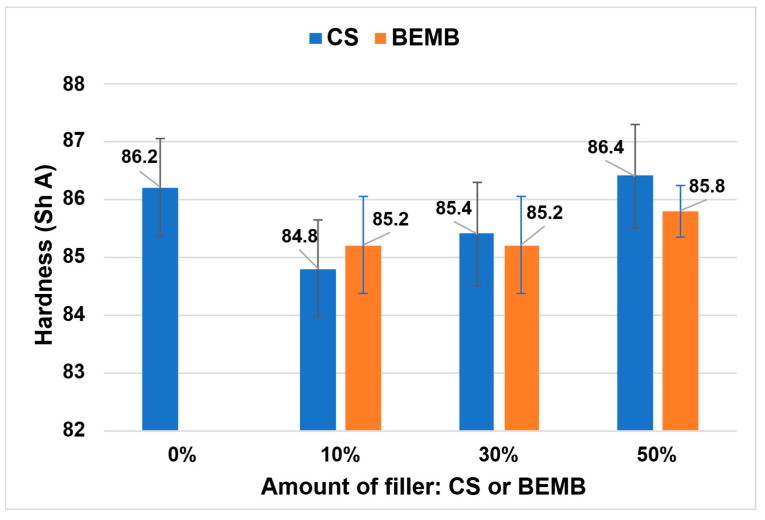
Hardness value of the obtained materials.

The addition of a flame-retardant filler resulted in a slight decrease in the hardness of the composite compared to the reference sample (see Figure 12). It is noteworthy that partial substitution of chalk (50 wt%) with CS or BEMB enabled the production of composites with hardness values comparable to the reference material.

In both series of tested composites—those containing CS and those with BEMB—a progressive increase in hardness was observed with increasing content of the flame-retardant filler. This effect was particularly pronounced in composites modified with CS, which exhibited greater increases in hardness compared to analogous samples containing BEMB.

The mechanical properties under static tensile testing were also examined for the materials received, and the numerical values are presented in Table 4.

Based on the data presented in the table above, partial replacement of chalk with flame-retardant fillers such as CS or BEMB leads to a reduction in tensile strength and elongation at break of the analyzed composites. As the content of these fillers increases, a significant deterioration in the aforementioned mechanical properties is observed.

When comparing the effects of CS and BEMB on mechanical performance, the results indicate that the use of CS results in a relatively smaller decline in mechanical parameters compared to BEMB. Particularly noteworthy trends were observed in the values of the elastic modulus. In composites containing CS, an increase in filler content correlates with an increase in elastic modulus values. In contrast, composites with BEMB exhibit an opposite trend, where higher filler content leads to a decrease in this parameter.

The reduction in the mechanical strength of the composites may be attributed to the poor compatibility between the filler and the polymer matrix.

The observed changes in elastic modulus values indicate that the incorporation of chitosan as a filler leads to increased stiffness of the composite, which is likely attributable to the specific characteristics of its chemical structure. In contrast, the addition of BEMB in high concentrations results in a reduction of the mechanical performance of the composite materials. This effect can be attributed to the limited dispersibility of BEMB and its weaker compatibility with the polymer matrix, as compared to composites containing chitosan as the filler.

## 3. Materials and Methods

### 3.1. Chemicals and Measurements

All commercially available chemicals and solvents obtained were of high quality and employed without further purification.

Dibutyl phosphate was purchased from Sigma-Aldrich (Darmstadt, Germany). The high-purity aluminium oxide hydroxide (boehmite) PURAL SB was made available for our research courtesy of the company Sasol, Brunsbüttel, Germany.

The PVC composites were formulated using the following reagents: poly(vinyl chloride) (PVC, Polanvil S-70, supplied by Anwil S.A., Polska), diisononyl phthalate (DINP, Sigma Aldrich) as a plasticizer, zinc stearate (Sigma Aldrich) as a thermal stabilizer, calcium carbonate (chalk, Sigma Aldrich) as a filler chitosan (CS) (low molecular weight, powder form; molecular weight 50,000–190,000 Da, viscosity 20–300 cP for 1 wt% solution in 1% acetic acid at 25 °C, soluble in dilute aqueous acids; Sigma-Aldrich) and BEMB as an ecological flame retardants.

The Fourier transform infrared (FT-IR) spectra were obtained with a Bruker Tensor 27 FTIR spectrometer (Ettlingen, Germany) applying the attenuated total reflectance (ATR) method. The samples were powder (BEM, BEMB, and CS) or formed by injection molding (PVC-based composites), and their spectra were recorded at room temperature, averaging 32 scans within the range of 600 to 4000 cm^−1^, with a resolution of 4 cm^−1^ in absorbance mode.

The Quanta 3D FEG scanning electron microscope (SEM) operating at an acceleration voltage of 20 kV, combined with energy dispersive X-ray spectroscopy (EDX) from FEI, USA, was employed to visualize the morphology and microstructure of materials, along with their chemical composition.

Differential scanning calorimetry (DSC) measurements were applied to a Netzsch DSC 204 calorimeter (Günzbung, Germany) in dynamic mode. DSC measurements were carried out utilizing aluminum pans featuring pierced lids, with a sample mass of approximately 5–10 mg, under a nitrogen atmosphere at a flow rate of 30 mL/min. Dynamic scans were performed at a heating rate of 10 °C/min within a temperature range of −50 to 550 °C.

TGA was carried out using a Netzsch STA 449 F1 Jupiter thermal analyzer (Selb, Germany) at temperatures from 30 to 1000 °C under a helium atmosphere (gas flow rate of 25 cm^3^/min) with a heating rate of 10 °C/min. Sample masses of about 10 mg were tested in open aluminum oxide crucibles, and as a reference, an empty crucible was employed.

The flammability of PVC-based composites was assessed using the horizontal burning method, under Method A UL94HB as specified in the European standard EN 60695-11-10:2014-02 [39]. This standardized procedure enables the determination of the material’s burning behaviour under controlled conditions. Test specimens were mounted horizontally in a metal clamp affixed to a tripod. A 50 W burner, inclined at an angle of 45°, was used to apply a flame to the free end of the sample for a duration of 30 s. Following flame exposure, the time required for the sample to self-extinguish was recorded. Alternatively, the sample could be extinguished manually after 15 s of free burning, allowing for the measurement of the burned length and the rate of combustion. The linear burning rate (v) was calculated using the following equation:

v = 60 × L/t(2)

where

v—linear burning rate, expressed in millimeters per minute (mm/min),
L—length of the burned part of the sample, in millimeters (mm),
t—time of combustion, in seconds (s).

The hardness of the composite samples was determined using the Shore A method, in accordance with the International Standard EN ISO 868:2003 [40]. Measurements were performed using a Zwick 7206/H04 hardness tester (Zwick Roell, Ulm, Germany). Hardness values were recorded 15 s after the indenter made contact with the sample surface, as specified by the standard.

Tensile properties of the composites were evaluated using a Zwick/Roell Z010 universal testing machine (Zwick Roell, Ulm, Germany), following the procedures outlined in the International Standard ISO 527-2:2012 [41]. Test specimens were cut from the prepared sheets and subjected to tensile loading at a constant crosshead speed of 100 mm/min.

### 3.2. Modification of Boehmite

A mixture of 50 mL dibutyl phosphate (47.2 g, 0.22 moles) and bismuth (4.19 g, 0.07 moles) in 250 mL of solvent toluene (T), xylene (K), or mesitylene (M) was heated to boiling temperature using an azeotropic distillation receiver for 24 h. After cooling, the crude product was filtered under reduced pressure, purified in toluene using a Soxhlet apparatus to remove trace impurities, and dried under reduced pressure at 65 °C.

### 3.3. Preparation of Composites

**Figure 13:**
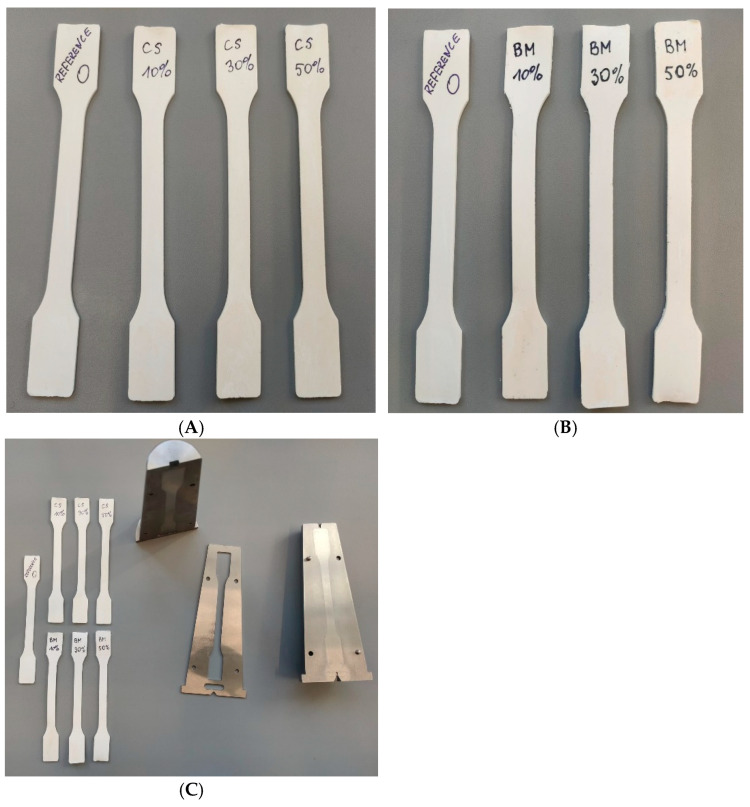
Photos of the samples: (**A**) chitosan derivatives, (**B**) BEMB derivatives, (**C**) samples with mold for micro-injection molding machine.

The preparation of dry blend-type mixtures began with introducing accurately weighed amounts of poly(vinyl chloride) (PVC) and a thermal stabilizer (zinc stearate) into the mixer. These components were mixed until the temperature reached approximately 50 °C. Next, a plasticizer (DINP)—preheated to 50 °C to prevent cooling of the entire mixture and to enable its rapid and uniform distribution within the polymer structure—was added to the system. After the plasticizer was introduced, mixing continued until a homogeneous consistency of the blend was achieved. During this stage, preliminary plasticization of the PVC occurred, which facilitated its subsequent integration with the remaining components. The next step involved the gradual addition of chalk and, depending on the sample type, an appropriate flame retardant (CS or BEMB). These ingredients were thoroughly mixed to ensure their even distribution within the polymer matrix and to prevent the formation of agglomerates. Mixing was carried out until the temperature of the mixture reached approximately 110 °C. The elevated temperature promoted better particle bonding and maintained a high level of homogeneity in the blend. As a result of the entire process, a dry powder product was obtained, ready for further processing. Next, using a laboratory microinjection molding machine, paddle-shaped specimens were produced for further testing. In this way, two series of composites were obtained, in which the filler (chalk) was partially replaced (10 wt%, 30 wt%, and 50 wt%) with chitosan or modified boehmite. These samples were labeled accordingly as CS 10%, CS 30%, CS 50%, BM 10%, BM 30%, and BM 50%. For better analysis of the results, a reference sample without any flame retardant was prepared in the same manner. In Figure 13, photos of the prepared samples are presented.

## 4. Conclusions

The synthesis of aluminium dibutyl phosphonate using various high-boiling aromatic solvents proved to be an efficient process, consistently yielding over 80% fine-grained product. ATR/FT-IR analysis confirmed that the reaction temperature significantly influenced the structural characteristics of the final materials.

TG analysis confirmed that the thermal decomposition of pure boehmite occurs in two distinct stages: the release of physically bound water and structural transformation into aluminium oxide. Modifying the material with dibutyl phosphonate significantly altered its thermal behaviour, reducing the residual mass and indicating the partial incorporation of organic components into its structure.

Thermogravimetric analysis revealed that the addition of chitosan to PVC composites resulted in a more significant reduction in thermal stability and residual mass compared to composites containing modified boehmite. The degradation process proceeded in multiple stages, a typical characteristic of PVC-based materials. The most intense changes occurred during the dehydrochlorination process and the breakdown of the inorganic fillers. Modified boehmite was found to be more thermally stable, leaving a higher solid residue and indicating its beneficial contribution to the composites’ fire resistance. Analysis of the FT-IR spectrum of the volatile decomposition products confirmed the presence of characteristic HCl and C–Cl bands, indicating PVC dehydrochlorination. At higher temperatures, the halogen compound and phthalate bands disappeared, and the CO_2_ and water signals became dominant, which confirms the multi-stage mechanism of PVC degradation.

In terms of flammability, the addition of chitosan was found to significantly reduce the rate of flame spread by more than 50%, compared to the reference sample. Conversely, increasing the content of modified boehmite increased the combustion rate, suggesting that phosphorus is less effective at retardation than nitrogen atoms in chitosan.

Analysis of the mechanical properties showed that partially replacing chalk with fire-retardant additives decreased tensile strength and elongation at break, with this effect being milder in the case of chitosan. Furthermore, CS additives increased the elastic modulus of the composites, thereby improving their stiffness; however, BEMB additives at higher concentrations decreased stiffness.

Based on the conducted experimental studies, it was demonstrated that among the analyzed eco-friendly flame retardants, chitosan exhibits the most favorable toxicological profile, as well as superior mechanical strength and flame inhibition properties, outperforming modified boehmite. The obtained results indicate the potential for chitosan to be used as a functional additive in the production of environmentally friendly cable insulation materials based on poly(vinyl chloride) composites.

## Figures and Tables

**Table 1:** SEM-EDX surface atom ratio of boehmite samples (BEM) and aluminum dibutyl phosphate samples (BEMB_T, K, M).

Sample	Element	OK	AlK	PK	CK	Total
BEM	wt%	56.71	43.29	-	-	100
	at%	68.84	31.16	-	-	100
BEMB_T	wt%	38.17	13.29	10.84	37.71	100
	at%	37.47	7.73	5.49	49.31	100
BEMB_K	wt%	33.12	5.10	14.59	47.19	100
	at%	31.09	2.84	7.07	59.00	100
BEMB_M	wt%	36.95	4.54	11.58	46.93	100
	at%	34.17	2.49	5.53	57.81	100

**Table 2:** TGA data of the boehmite and its modification.

Sample	*T*_1_ ^1^ (°C)	*T*_5_ ^2^ (°C)	*T_max_* ^3^ (°C)	Residual Mass (%)
BEM	110	279	81; 423	78.33
BEMB	238	273	294; 426	44.67

^1,2^ The temperatures of 1% and 5% mass loss, respectively; ^3^ The temperatures of the maximum rate of mass loss.

**Table 3:** TG data on the prepared materials.

Sample	*T*_1_ ^1^ (°C)	*T*_5_ ^2^ (°C)	*T_max_* ^3^ (°C)	Residual Mass (%)
Reference	229	260	292; 448; 533; 594; 673	22.88
CS 10%	229	259	296; 446; 520; 589; 672	22.46
CS 30%	213	248	285; 440; 538; 597; 670	17.41
CS 50%	211	244	277; 442; 539; 591; 666	12.80
BM 10%	211	245	257; 296; 449; 554; 595; 669	20.88
BM 30%	213	246	256; 290; 303; 450; 582; 690	20.54
BM 50%	225	249	256; 287; 302; 436; 528; 658	19.08

^1,2^ The temperatures of 1% and 5% mass loss, respectively; ^3^ The temperatures of the maximum rate of mass loss.

**Table 4:** Mechanical properties of the materials received.

Sample	Tensile Strength (MPa)	Modulus of Elasticity (MPa)	Elongation at Break (%)
Reference	13.36 ± 0.26	7.41 ± 0.15	314.85 ± 10.23
CS 10%	11.39 ± 0.67	6.84 ± 0.59	262.43 ± 27.20
CS 30%	9.50 ± 0.70	7.36 ± 0.62	203.69 ± 14.00
CS 50%	8.10 ± 0.36	7.92 ± 0.43	168.92 ± 21.65
BM 10%	11.50 ± 0.49	6.09 ± 0.26	279.21 ± 23.07
BM 30%	8.81 ± 0.23	5.68 ± 0.16	191.29 ± 15.86
BM 50%	7.04 ± 0.77	5.27 ± 0.24	158.35 ± 24.17

## Data Availability

All data are available from the corresponding author upon reasonable request.
